# Biomechanical comparison of different bilateral percutaneous vertebroplasty in treating osteoporotic vertebral compression fractures: finite element analysis

**DOI:** 10.3389/fsurg.2025.1691126

**Published:** 2025-11-06

**Authors:** Weihua Yang, Jing Chen, Bei Lin, Eryou Feng

**Affiliations:** 1 Department of Arthrosis Surgery, Fujian Medical University Union Hospital, Fuzhou, China; 2 Department of Rehabilitation Medicine, Anxi County Hospital, Quanzhou, China

**Keywords:** osteoporosis, vertebral compression fracture, vertebroplasty, finite element analysis, bone cement distribution

## Abstract

**Purpose:**

This study aimed to analyze the biomechanical effects of two bone cement injection techniques by establishing a finite element model of osteoporotic vertebral compression fractures.

**Methods:**

CT data from a healthy male volunteer were used to construct a three-dimensional finite element model of the L1–L3 vertebrae. A simulated vertebral compression fracture was created at L2, followed by virtual vertebroplasty using two cement distribution patterns: the vertical group (VG) and the inclined group (IG). Stress distribution, maximum von Mises stress in the vertebrae and intervertebral discs, and the maximum displacement of L2 were compared between the two groups.

**Results:**

In the L2 vertebra, the maximum stress in the VG is reduced under all six loading conditions. VG showed reduced maximum stress in the L1 vertebra during extension, left bending, and left/right rotation. For the L3 vertebra, VG achieved the lowest maximum stress under all loading conditions. In the L1–L2 intervertebral disc, VG resulted in lower maximum stress than IG during flexion, extension, and lateral bending. while in the L2–L3 disc, VG produced the lowest maximum stress under all six conditions. Furthermore, under flexion and extension, the maximum displacement of L2 was smaller in VG compared with IG.

**Conclusions:**

The vertical cement distribution pattern effectively reduces vertebral and intervertebral disc stress and provides greater stability of the fractured vertebra compared with the inclined distribution pattern.

## Introduction

Osteoporosis (OP) is a systemic skeletal disorder characterized by decreased bone mass and deterioration of bone microarchitecture, leading to increased bone fragility and susceptibility to fractures (1). Globally, approximately 200 million individuals are affected by osteoporosis, presenting with varying degrees of pain, spinal deformity, fragility fractures, and reduced muscle strength (2, 3). The decline in bone mass significantly elevates fracture risk, particularly in the vertebrae, hip, and wrist. Among these, osteoporotic vertebral compression fractures (OVCFs) are the most prevalent (4). With the continuing aging of the global population, an estimated 1.4 million OVCF cases occur annually, and the incidence among individuals aged over 50 years ranges from 10% to 25% (5, 6). OVCF has thus emerged as a major public health concern, imposing a substantial healthcare burden, impairing the quality of life of elderly individuals, and increasing mortality rates (7).

**Table 1:** Material properties of finite element analysis models.

Parts	Young modulus (MPa)	Poisson ratio	Element type	References
Normal cortical bone	12,000	0.3	C3D8	Li et al. (25)
Osteoporotic cortical bone	8,040	0.3	C3D8	Li et al. (25)
Normal cancellous bone	132	0.2	C3D8	Li et al. (25)
Osteoporotic cancellous bone	34	0.2	C3D8	Zhou et al. (1)
Normal endplate	1,000	0.4	C3D8	Zhou et al. (1)
Osteoporotic endplate	670	0.4	C3D8	Liang et al. (18)
Intervertebral disc				Liang et al. (18)
Bone cement (PMMA)	3,000	0.4	C3D8	Zuo et al. (3)
Nucleus pulposus	0.2	0.49		Liang et al. (18)
Annulus fibrosus	4.2	0.45		Zhao et al. (13)
ALL	20	0.3	spring	
PLL	20	0.3	spring	
LF	19.5	0.3	spring	
SSL	15	0.3	spring	
ISL	12	0.3	spring	
CL	7.5	0.3	spring	
ITL	50	0.3	spring	

ALL, anterior longitudinal ligament; PLL, posterior longitudinal ligament; LF, ligamentum flavum; SSL, supraspinal ligament; ISL, interspinous ligament; ITL, intertransverse ligament; CL, capsular ligament.

In the early stages following OVCF, conservative management involving bed rest is typically adopted. Once the fracture site stabilizes after 4–6 weeks of immobilization, gradual ambulation may be initiated (8). However, conservative treatment is often associated with complications such as bone loss, pressure ulcers, hypostatic pneumonia, and muscle atrophy (6, 9). Moreover, it is unsuitable for patients with severe comorbidities, unstable fractures, or nonunion (10, 11). In recent years, percutaneous vertebroplasty (PVP) has gained wide clinical acceptance for the management of OVCF due to its advantages of broad applicability, minimal invasiveness, and procedural simplicity (12). During PVP, a puncture needle is inserted into the affected vertebra under imaging guidance, and bone cement is injected into the vertebral body. The cement rapidly polymerizes within the vertebra, thereby stabilizing the fracture, restoring vertebral height, and alleviating pain. PVP has demonstrated substantial efficacy in treating painful osteoporotic fractures, with 80%–90% of patients experiencing rapid pain relief and functional improvement postoperatively (10, 13).

The intravertebral distribution of bone cement has been shown to affect the stress profile of both the treated and adjacent vertebrae (14). Several studies have indicated that bilateral cement distribution more effectively relieves pain and restores vertebral height compared with unilateral distribution (15). However, on the sagittal plane, variations in cement distribution arise due to differences in injection trajectory and volume. Zhang et al. (16) classified cement distribution into four types based on endplate contact and found that these patterns influenced intervertebral disc degeneration. Building upon this, we categorized cement distribution into two major sagittal configurations based on postoperative imaging analyses: (1) a vertical distribution, in which the cement spreads evenly and remains parallel to the vertebral endplate, and (2) an inclined distribution, in which the cement extends obliquely from the anteroinferior to the posterosuperior portion of the vertebral body. Cement distribution is influenced by both the orientation of the fracture line and the puncture angle during injection (17). Uniform cement spread parallel to the vertebral axis ensures even mechanical support, whereas cement aligned along the puncture needle tends to create an angular, asymmetric distribution. Previous studies have suggested that such asymmetry may lead to insufficient mechanical support and abnormal stress transmission to adjacent segments, thereby predisposing to refracture or adjacent vertebral fractures (18).

Although existing clinical studies have identified cement distribution as a key determinant of postoperative outcomes (19), most rely on cohort analyses and lack biomechanical validation. Since the 1970s, finite element analysis (FEA) has become a cornerstone of spinal biomechanics research owing to its precision, individualization, and cost-effectiveness (20). Prior investigations have demonstrated that bilateral vertebroplasty achieves superior stress balance across vertebrae (21). However, under bilateral injection conditions, the specific biomechanical impact of different cement distribution patterns—particularly vertical (symmetric) vs. inclined (oblique) configurations—remains poorly understood.

Therefore, the present study systematically compared the biomechanical characteristics of vertical and inclined cement distribution patterns in bilateral PVP using the finite element method. By analyzing stress distribution and vertebral stability, this study aims to elucidate the mechanical implications of these configurations and provide a theoretical foundation for optimizing clinical injection techniques.

## Materials and methods

### Spinal data acquisition and construction of L1–L3 finite element model

A healthy adult male volunteer with no history of spinal disease or surgery was selected. Computed tomography (CT) scans of the L1–L3 vertebrae were obtained with a slice thickness of 0.5 mm. The data were imported into Mimics 21.0 (Materialise, Leuven, Belgium) in Digital Imaging and Communications in Medicine (DICOM) format to reconstruct a three-dimensional lumbar spine model through grayscale adjustment and threshold segmentation.

Subsequently, Geomagic Wrap 2021 (Geomagic, USA) was used for noise reduction, surface smoothing, contour editing, mesh optimization, and surface fitting. The processed model was further segmented and assembled in SolidWorks 2021 (Dassault Systèmes, USA), where the upper and lower endplates, articular cartilage, and intervertebral disc structures were established. The cortical bone thickness was set at 1.5 mm (22). The intervertebral disc was modeled as consisting of a nucleus pulposus and an annulus fibrosus, with the nucleus pulposus occupying 40% of the total disc area (23). The completed model was saved in SLDPRT format for subsequent analysis.

### Construction of compression fracture model

**Figure 1:**
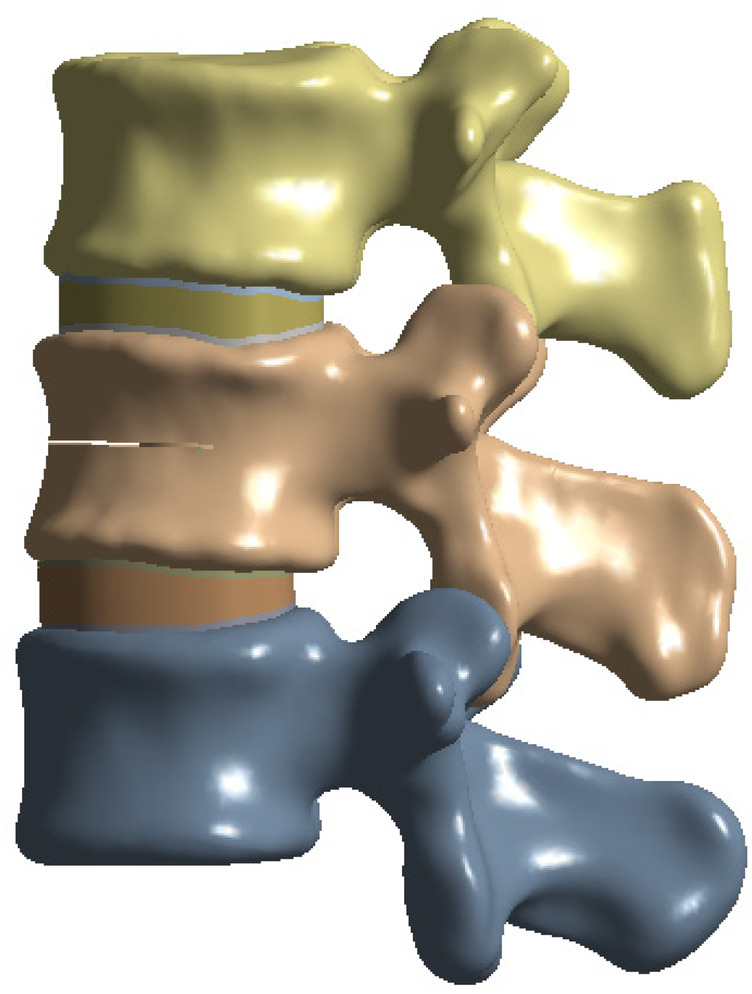
L2 vertebral fracture model.

To simulate osteoporotic bone, the elastic modulus was reduced based on previously published data (23, 24). The L2 vertebral compression fracture model was created by introducing a 0.5 mm horizontal fracture line through the anterior cortex of the vertebral body, extending posteriorly to the middle column (25). The approximate depth, width, and height of the fracture line were 22.5 mm, 42.5 mm, and 0.5 mm, respectively (26) (Figure 1).

### Establishment of bone cement model

**Figure 2:**
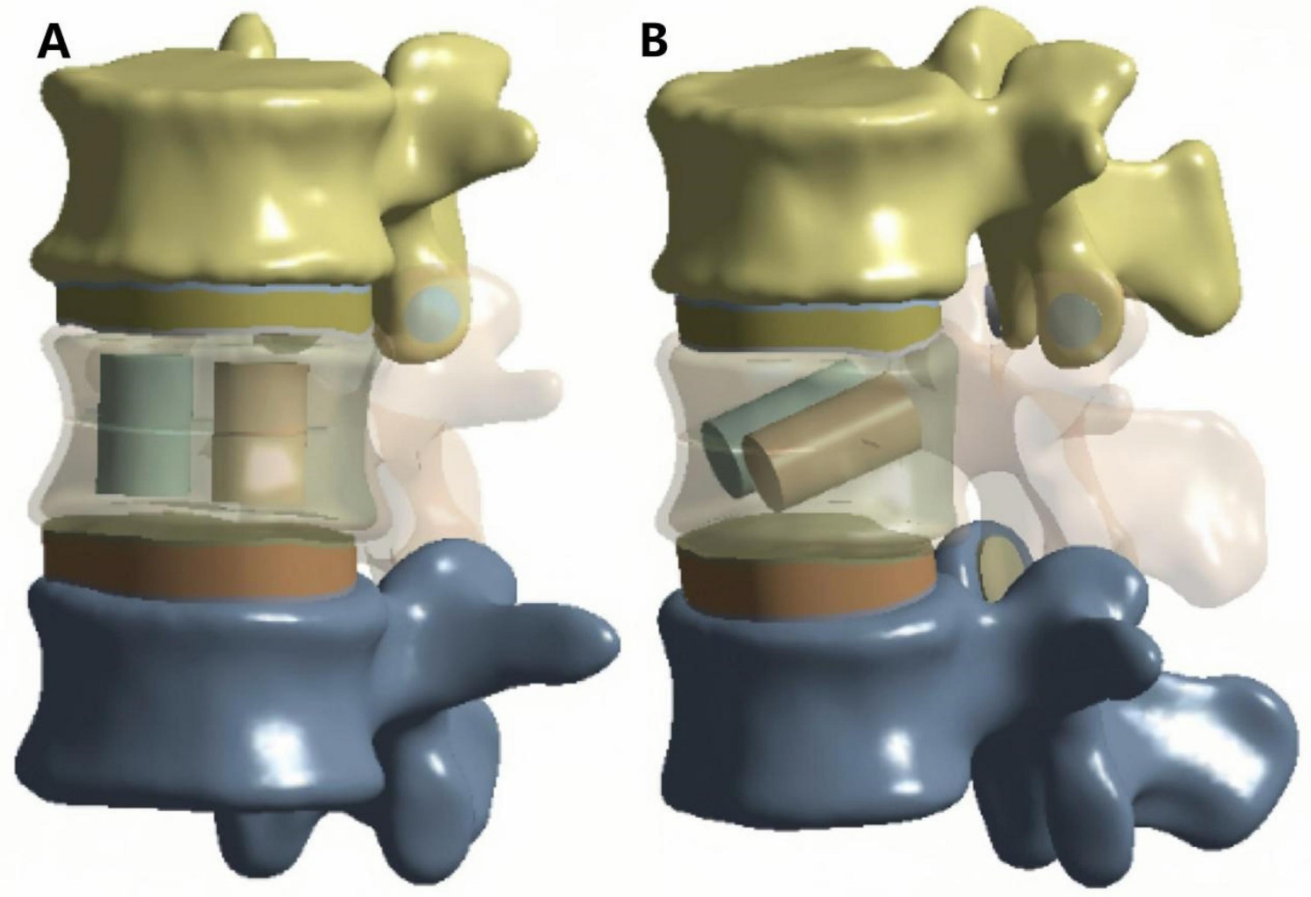
Bone cement distribution map. **(A)** VG, vertical group. **(B)** IG, incline group.

Two cylindrical regions were used to simulate bone cement distribution within the fractured vertebra. Based on previous studies, the total cement volume was set to 4 ml (1). For the vertical group (VG), two equal-volume cylinders were positioned vertically and symmetrically on both sides of the fracture (Figure 2A). For the inclined group (IG), two equal-volume cylinders were placed obliquely on both sides of the vertebral body (Figure 2B).

### Construction of PEA model

Finite element analysis (FEA) was performed using ANSYS 19.0 (ANSYS Inc., USA). The complete three-dimensional model consisted of cortical bone, cancellous bone, bone cement, upper and lower endplates, annulus fibrosus, nucleus pulposus, and articular cartilage. Ligamentous structures, including the anterior longitudinal ligament (ALL), posterior longitudinal ligament (PLL), ligamentum flavum (LF), interspinous ligament (ISL), supraspinous ligament (SSL), capsular ligament (CL), and intertransverse ligament (ITL), were also incorporated.

**Figure 3:**
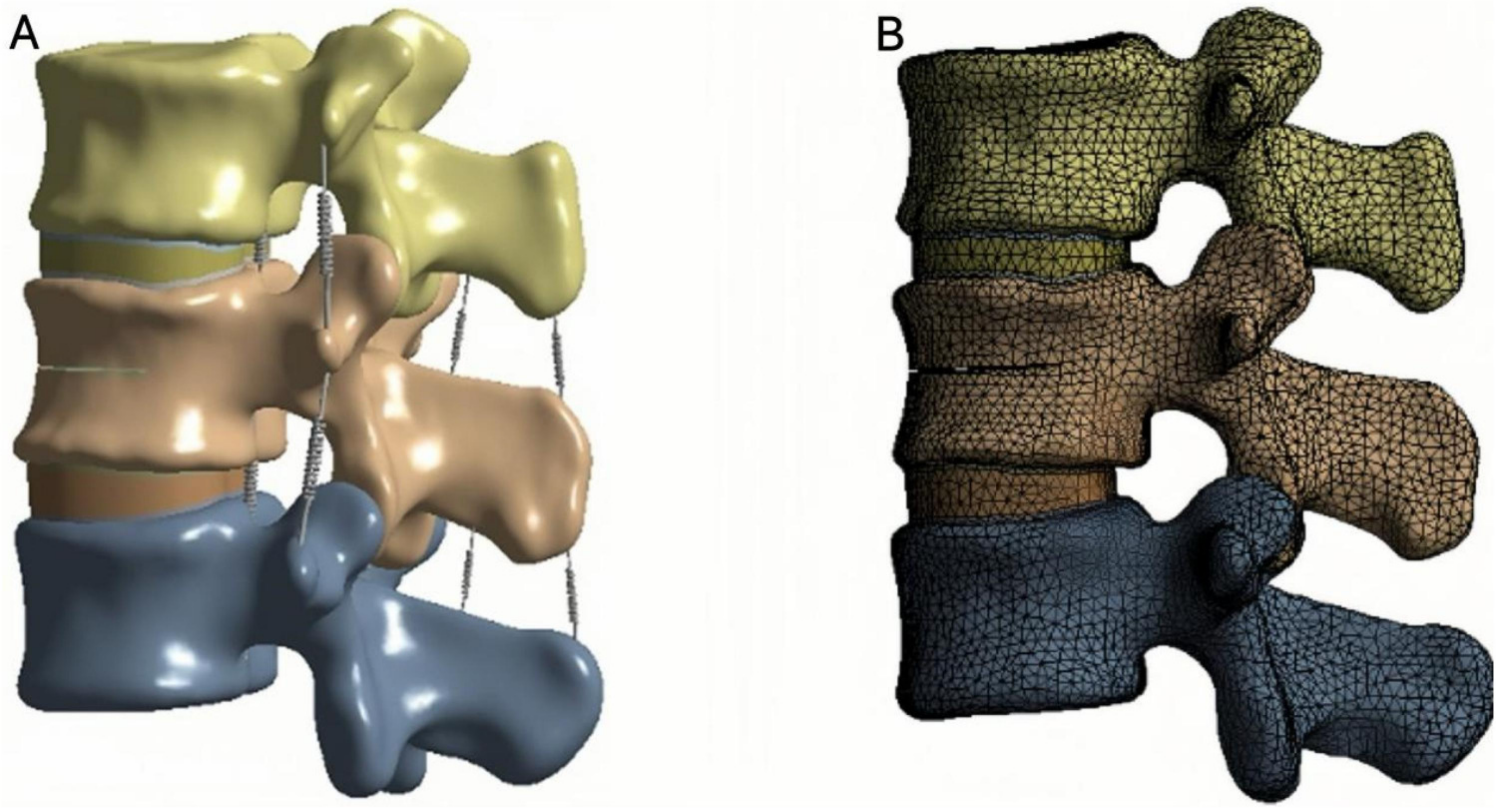
**(A)** L1–L3 finite element analysis model. **(B)** Mesh division of finite element model.

The cortical bone, cancellous bone, bone cement, endplates, intervertebral discs, and articular cartilage were defined as linear elastic isotropic materials, while the ligaments were modeled as hyperelastic elements that bear only tensile loads (27, 28) (Figure 3A; Table 1).

The endplates, cancellous bone, and bone cement were meshed with an element size of 2 mm; the cortical bone with 3 mm; the intervertebral disc (nucleus pulposus and annulus fibrosus) with 1.2 mm; and the articular cartilage with 0.8 mm (13, 29). Meshes, nodes, and elements were automatically generated by the software. The interfaces between the endplates and vertebral bodies, between endplates and discs, and between articular cartilage and bone were bonded to prevent relative motion (Figure 3B).

### Finite element analysis

**Figure 4:**
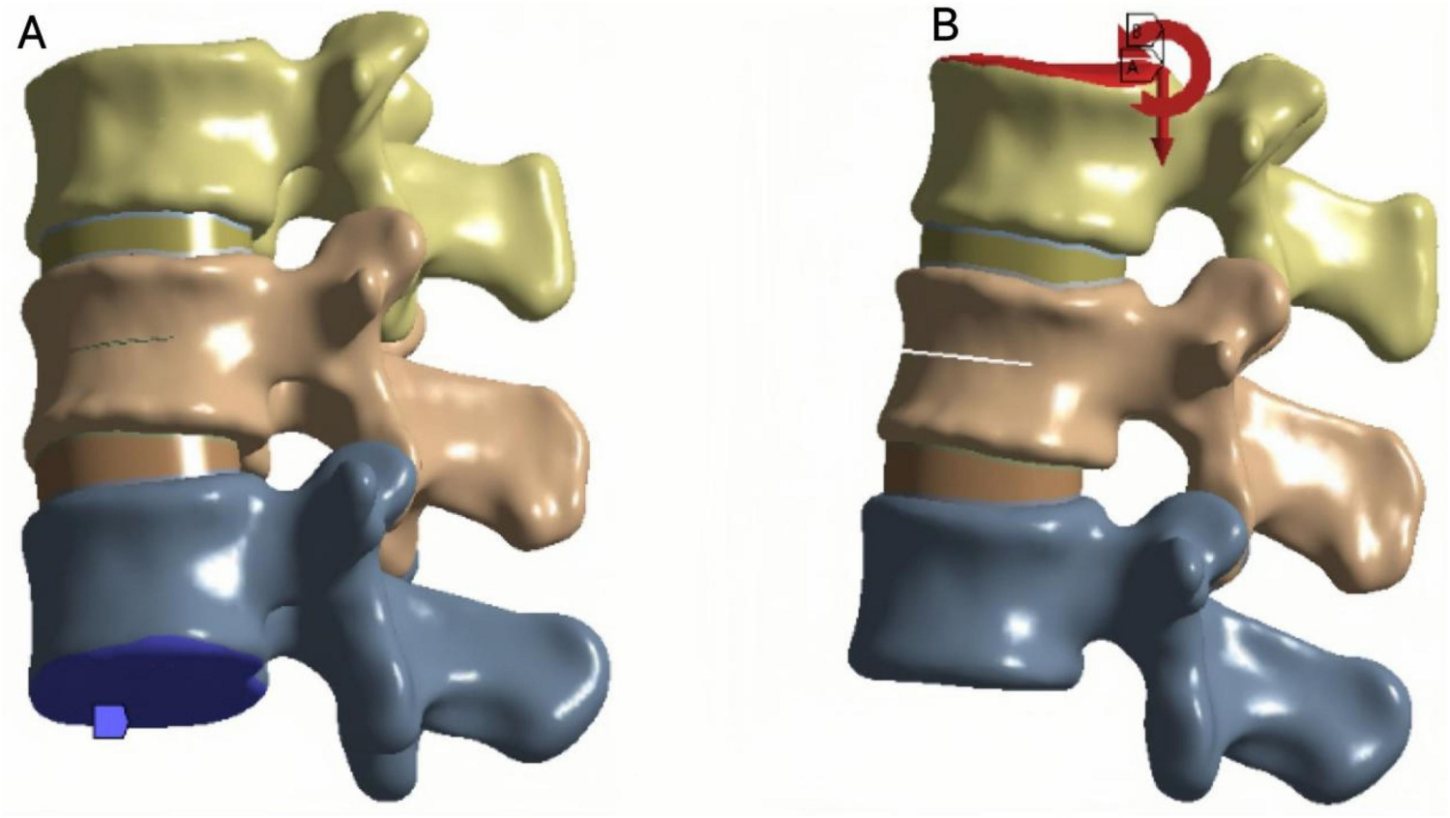
**(A)** Fix the lower surface of L3. **(B)** Apply a downward force of 500 N to the upper surface of L1 while applying a torque of 7.5 N.m.

To ensure boundary stability, the inferior surface of the L3 vertebra was fixed in all directions. A vertical compressive load of 500 N was applied to the superior surface of L1 to simulate the physiological load during upright posture (Figure 4A). In addition, a moment of 7.5 N·m was applied to reproduce six loading conditions of the lumbar spine: flexion, extension, left and right lateral bending, and left and right axial rotation (21) (Figure 4B).According to the three-column spinal load theory, 85% of the load was distributed to the anterior and middle columns, and 15% to the posterior column (25).

### Observation indicators

The magnitude and distribution of von Mises stress within each vertebra and intervertebral disc were calculated. The maximum displacement of the L2 vertebra was also measured. Von Mises stress was used to evaluate localized stress concentration and potential damage risk, whereas maximum displacement reflected the overall stability and deformation behavior of the spinal segment.

### Mesh convergence verification of finite element models

A mesh convergence study was conducted to verify the numerical results. Four models with progressively coarser element sizes in the L1–L3 segments (element sizes of 2.0, 2.5, 3.0, and 3.3 mm) were generated, and the maximum stress was evaluated for each. The analysis showed that the difference in maximum stress between the 2.5 mm and 3.0 mm meshes was less than 5%, meeting the convergence criterion. Considering that the 3.0 mm mesh ensures acceptable accuracy while substantially improving computational efficiency, it was selected as the final mesh size for the vertebral region (Table 2).

**Table 2:** The mesh convergence test.

Mesh Size (mm)	Number of elements	% Change in peak von Mises pressure
2	3,68,009	–
2.5	2,11,085	<5%
3	1,42,991	<5%
3.3	1,19,998	>5%

### Model verification

**Figure 5:**
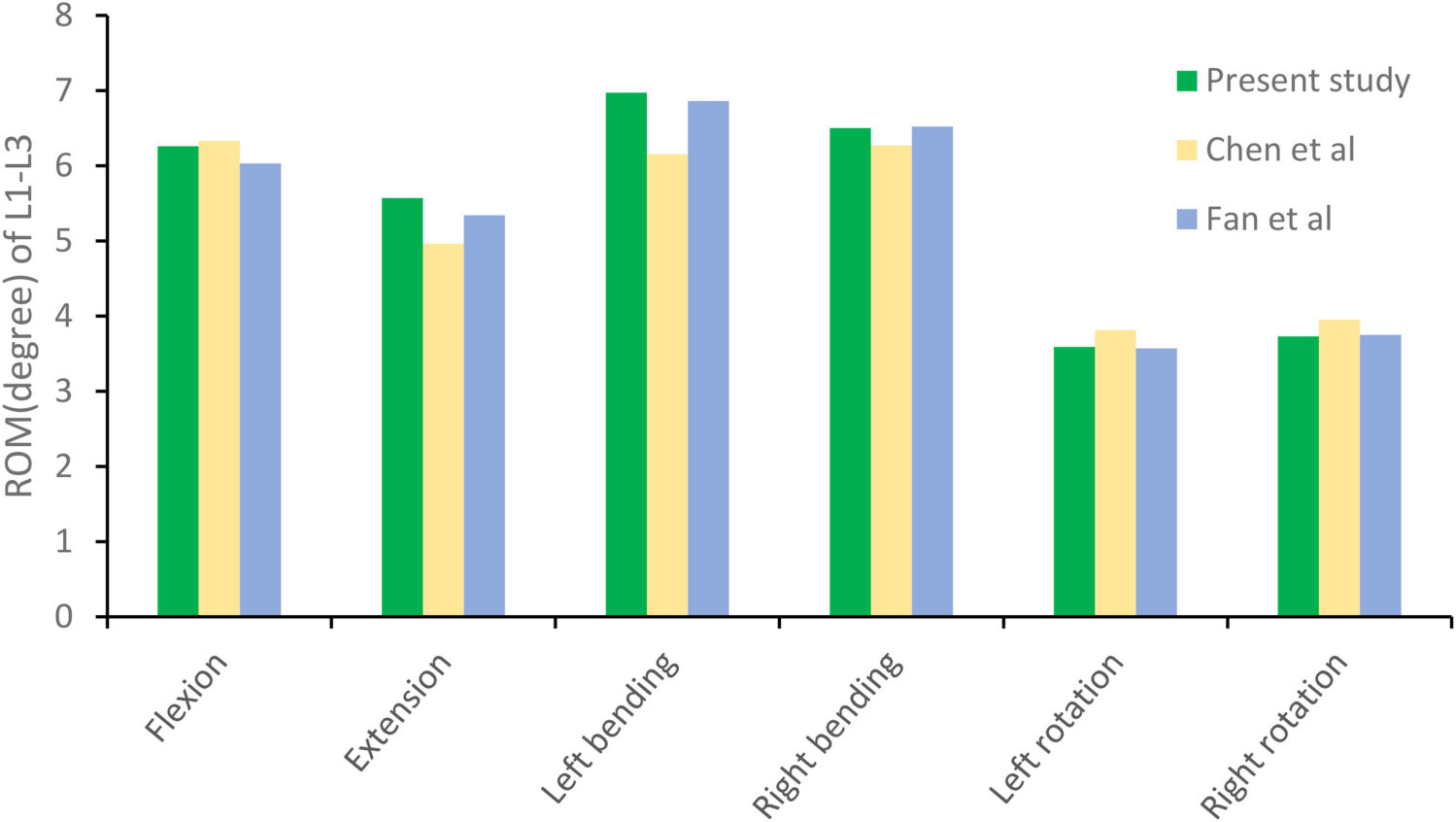
The range of motion (ROM) of the lumbar spine model in this study was compared with previously reported values.

The L1–L3 finite element model was validated under the same loading conditions by comparing the predicted range of motion (ROM) with data reported in previous biomechanical studies (30, 31). The simulated ROM values were consistent with those from prior experimental investigations (Figure 5), confirming the rationality, accuracy, and applicability of the constructed model for subsequent biomechanical simulations.

## Results

### The magnitude of von Mises stress of the L2 vertebral body

**Figure 6:**
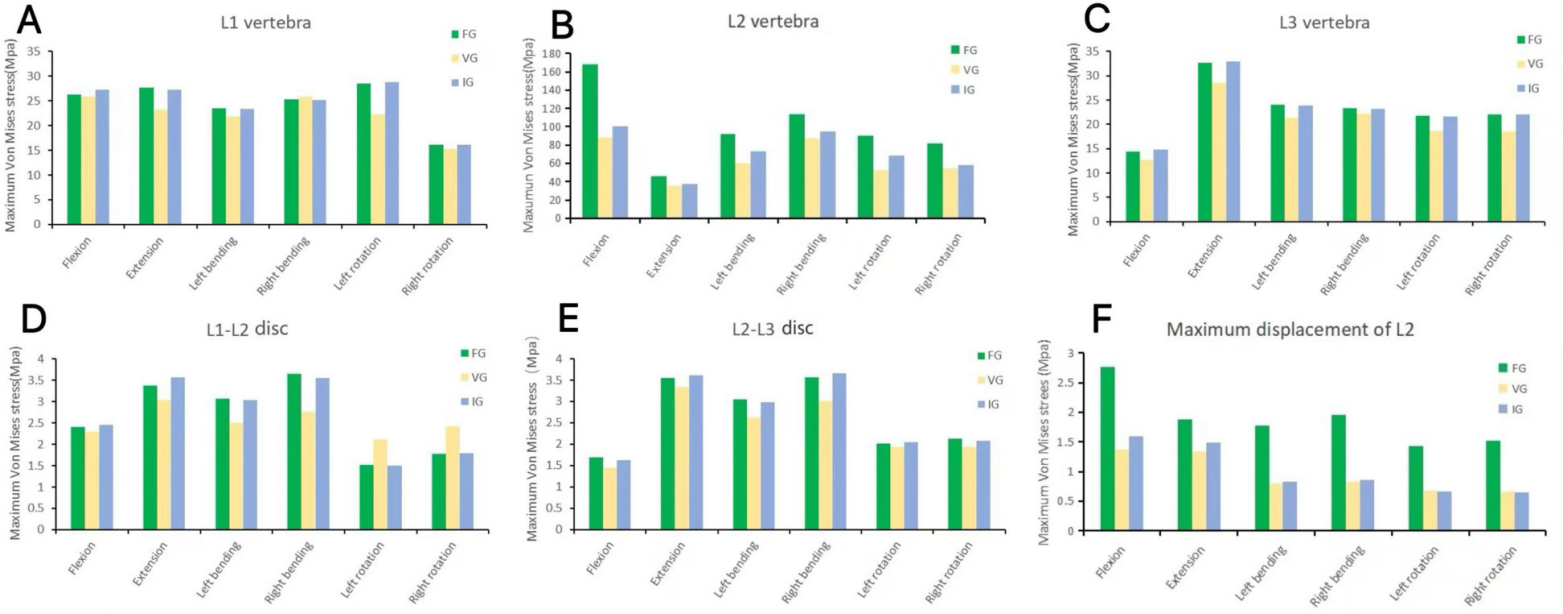
The maximum von Mises stress in the vertebrae **(A–C)** and intervertebral discs **(D,E)**, and the displacement of L2 **(F)**. FG, fracture group; IG, incline group; VG, vertical group.

**Figure 7:**
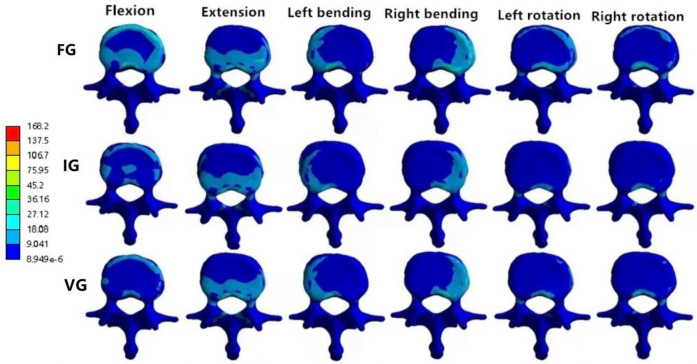
The distributions of the von Mises stress on L2. The distributions of the von Mises stress on L2 in FG, IG, and VG under flexion, extension, left/right bending, and left/right torsion.

In the L2 vertebra, the vertical group (VG) demonstrated a consistent reduction in maximum von Mises stress compared with the inclined group (IG) and fracture group (FG) under all six loading conditions. The maximum stresses in the IG were 99.99, 37.47, 72.80, 95.01, 68.57, and 58.14 MPa, respectively, whereas those in the VG were reduced to 88.21, 35.83, 60.15, 87.27, 52.81, and 54.05 MPa (Figures 6, 7).

### The magnitude of von Mises stress of L1 and L3 vertebral bodies

**Figure 8:**
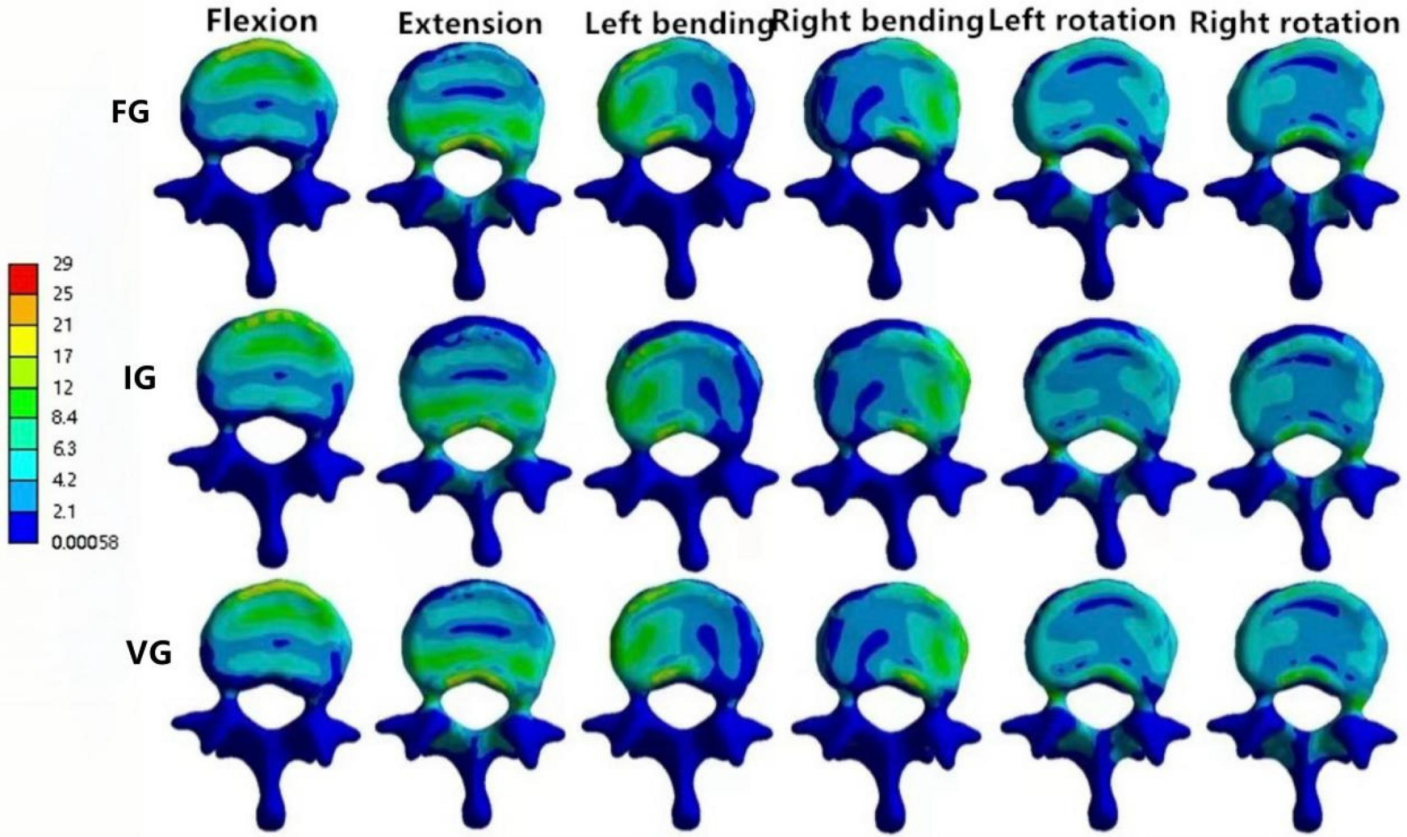
The distributions of the von Mises stress on L1. The distributions of the von Mises stress on L1 in FG, IG, and VG under flexion, extension, left/right bending, and left/right torsion.

**Figure 9:**
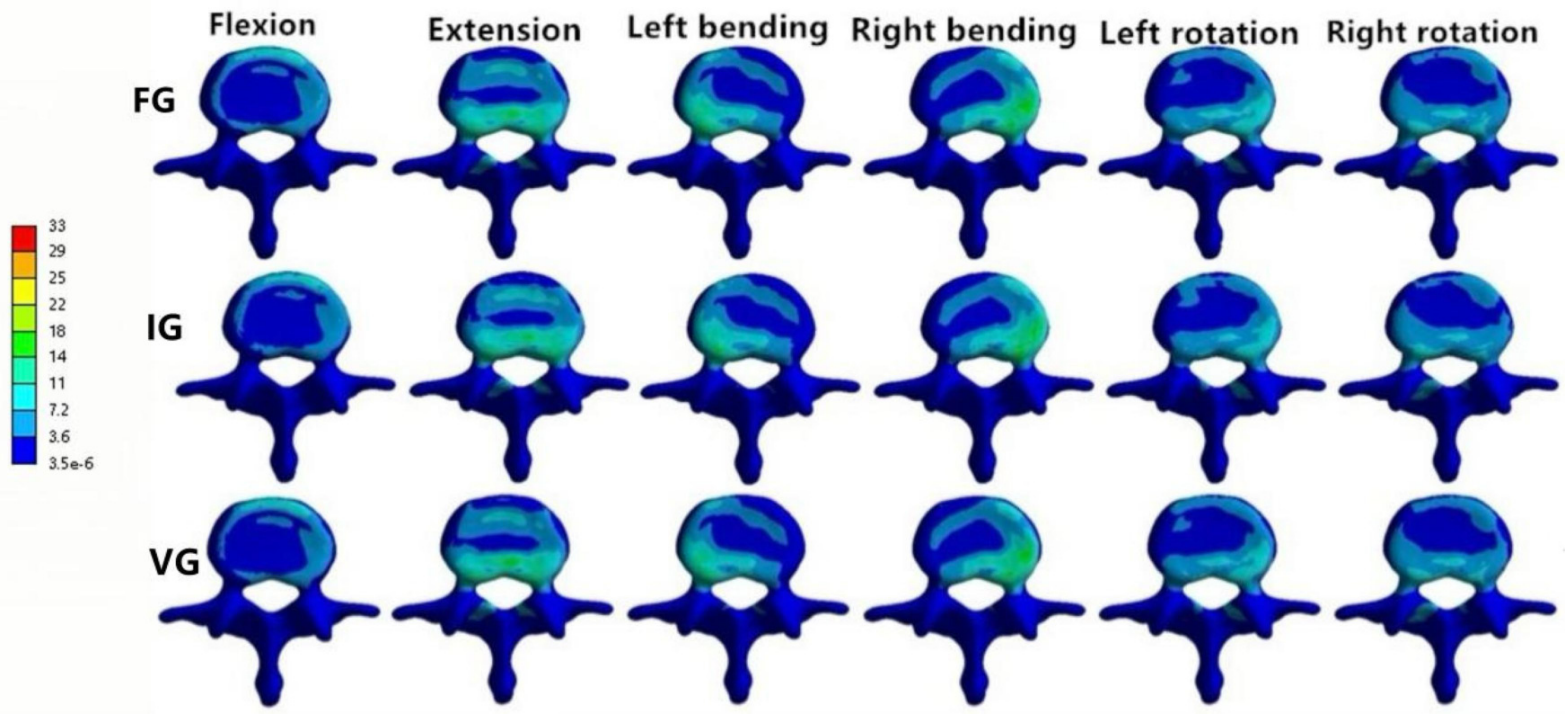
The distributions of the von Mises stress on L3. The distributions of the von Mises stress on L3 in FG, IG, and VG under flexion, extension, left/right bending, and left/right torsion.

In the L1 vertebra, the VG exhibited lower maximum stress than both the FG and IG during extension, left lateral bending, and left/right axial rotation, whereas the values during flexion and right bending were comparable among the three groups. Under the six loading conditions, the maximum stresses in the IG were 27.23, 27.28, 23.40, 25.19, 28.73, and 16.08 MPa, respectively; in contrast, the VG showed reduced values of 25.81, 23.24, 21.83, 25.83, 22.19, and 15.33 MPa.In the L3 vertebra, the VG demonstrated a marked decrease in maximum stress compared with both the FG and IG under all loading conditions (Figures 6, 8, 9).

### Magnitude of von Mises stress of intervertebral discs

**Figure 10:**
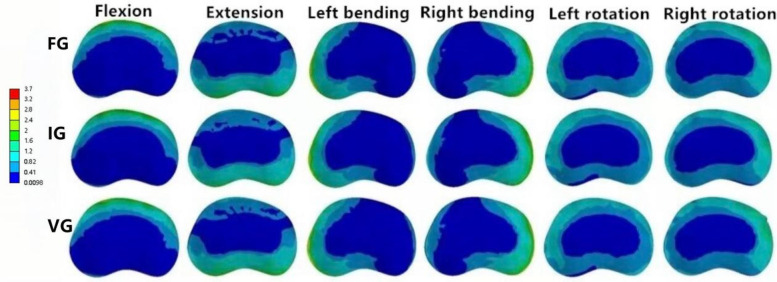
The distributions of the von Mises stress on the L1–L2 disc. The distributions of the von Mises stress on the L1–L2 disc in FG, IG, and VG under flexion, extension, left/right bending, and left/right torsion.

In the L1–L2 intervertebral disc, the VG exhibited lower maximum von Mises stress than the FG and IG during flexion, extension, and left/right bending, while slightly higher stress values were observed during left/right axial rotation (Figure 10).

**Figure 11:**
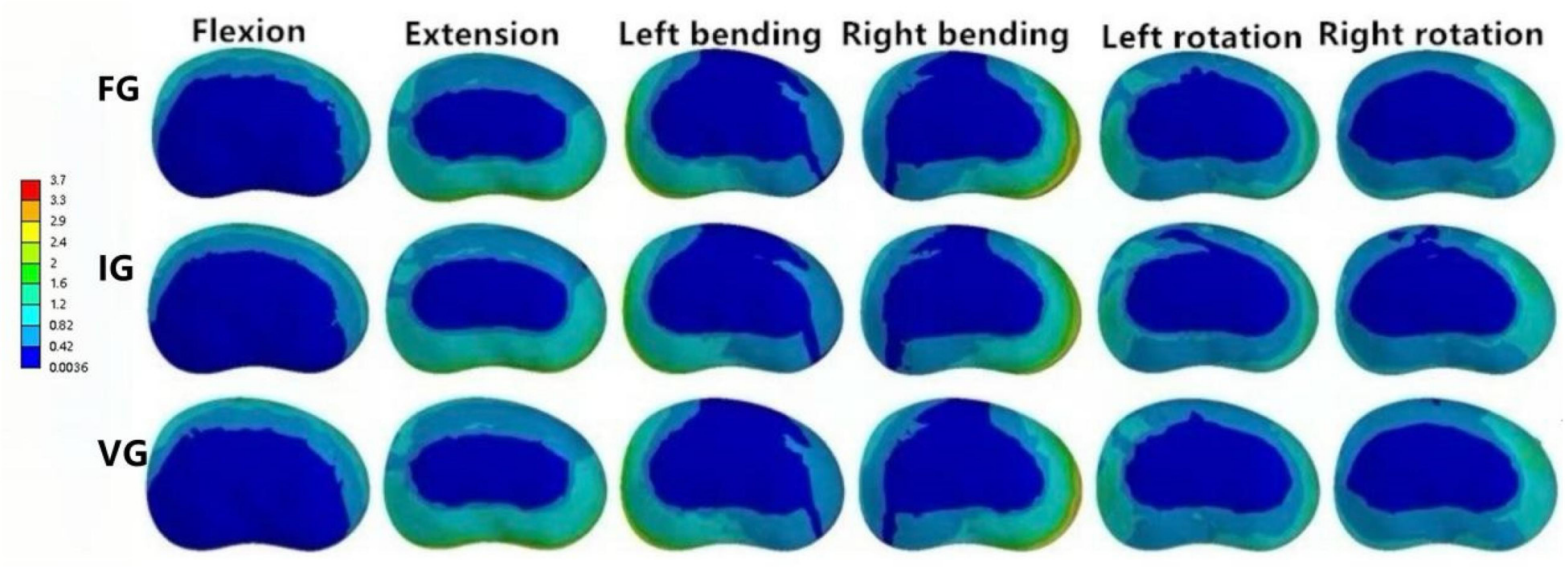
The distributions of the von Mises stress on the L2–L3 disc. The distributions of the von Mises stress on the L2–L3 disc in FG, IG, and VG under flexion, extension, left/right bending, and left/right torsion.

In the L2–L3 intervertebral disc, the VG model exhibited lower disc stress than the FG and IG models under all conditions (Figure 11).

### The maximum displacement of L2

**Figure 12:**
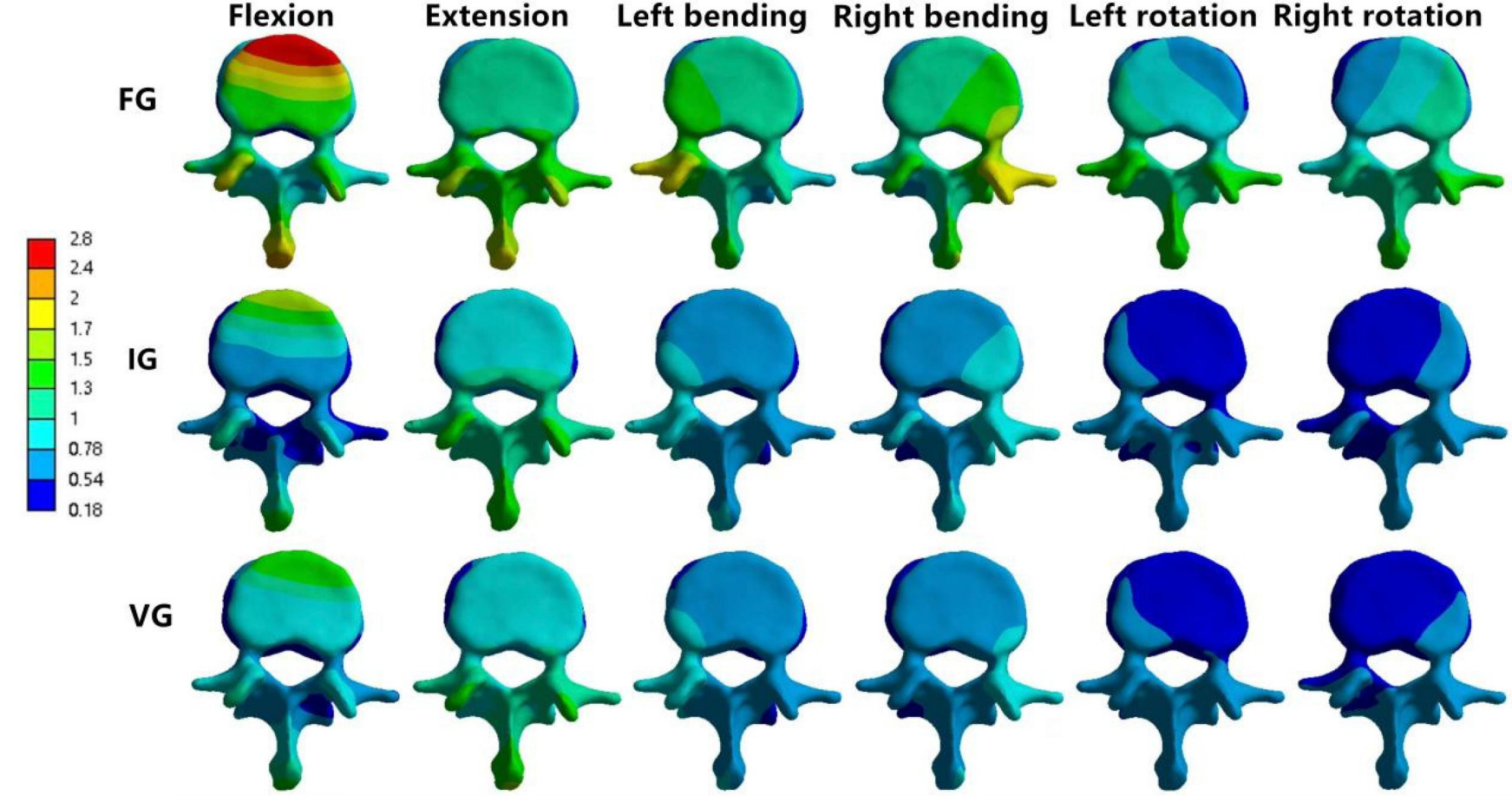
The maximum displacement of L2. The maximum Displacement of L2 in FG, IG, and VG under flexion, extension, left/right bending, and left/right torsion.

Compared with the FG, both the IG and VG models demonstrated reduced maximum displacement of the L2 vertebra under all loading conditions. Under flexion and extension, the VG exhibited lower displacement values than the IG, indicating improved stability. During left/right bending and left/right rotation, the displacements of the VG and IG were approximately equivalent (Figure 12).

## Discussion

Percutaneous vertebroplasty (PVP) has become one of the primary treatment options for osteoporotic vertebral compression fractures (OVCFs) owing to its proven clinical efficacy (32). However, as the procedure has gained popularity, reports of complications such as residual pain, secondary fractures, and loss of vertebral height have also increased (9, 33). Previous studies have indicated that both the distribution pattern and the volume of bone cement are key biomechanical factors associated with these complications (34). Accordingly, this study investigated the biomechanical effects of bone cement distribution using finite element analysis.

Several studies have demonstrated that the distribution pattern and injection volume of bone cement significantly influence the risk of refracture following PVP (35). In the present study, the maximum von Mises stress of the L2 vertebral body was markedly lower in the vertical group (VG) than in the other two models, suggesting that a uniform and symmetric cement distribution more effectively balances stress within the vertebra. In contrast, an inclined cement distribution results in asymmetric load transmission and stress concentration. Zhou et al. (36) reported that asymmetric cement distribution increases the likelihood of refracture in the augmented vertebra, while Wu et al. (34) found that symmetric cement distribution provides better stabilization of the fractured vertebra and reduces micromotion of the trabeculae, thereby alleviating pain and preserving vertebral height.

It is worth noting that cement volume is another critical determinant of postoperative biomechanics. To isolate the independent effect of distribution morphology, the cement volume in this study was standardized at 4 ml based on prior research (1, 34). Nonetheless, a potential interaction between cement volume and distribution pattern must be considered. Rohlmann et al. (37) demonstrated that cement volume strongly influences the maximum stress borne by the vertebrae. We speculate that at smaller volumes, the cement may fail to form an adequate internal support structure, thereby diminishing the benefits of favorable distribution patterns such as the symmetry seen in VG. Conversely, excessive cement volumes may increase vertebral stiffness, potentially leading to stress shielding and overloading of adjacent vertebrae. Future studies should therefore explore a broader range of cement volumes (e.g., 2–6 ml) in combination with different distribution patterns to identify optimal biomechanical parameters for specific clinical situations.

The displacement of the injured vertebra reflects the stability of the augmented segment (1). In this study, both the VG and IG models exhibited substantially reduced displacement compared with the fracture model (FG) under all six loading conditions, indicating that cement augmentation effectively enhances vertebral stability. Furthermore, the VG exhibited lower displacement values than the IG under flexion, extension, and lateral bending, suggesting that vertical cement distribution more effectively limits spinal micromotion and restores segmental stability.

An increased load on adjacent vertebrae following augmentation is a major cause of subsequent fractures (38). Nagaraja et al. (39) reported that bone cement reinforcement can double the incidence of fractures in the vertebra immediately superior to the treated segment. In the current study, the VG exhibited reduced maximum stress in the L1 vertebra during flexion, extension, and rotation compared with the other groups. Similarly, in the L3 vertebra, the VG showed the lowest maximum stress across all loading conditions. These findings suggest that vertically distributed bone cement restores the load-bearing capacity of the treated vertebra while reducing axial load transmission to adjacent levels, thereby potentially lowering the risk of refracture. However, the VG demonstrated slightly higher stress values during lateral bending than the IG, implying that excessive lateral bending movements should be avoided during postoperative rehabilitation to minimize stress transfer to adjacent segments.

Rohlmann et al. (37) found that bone cement infiltration can increase intradiscal pressure by approximately 20%. Zhao et al. (40) further reported that elevated intradiscal pressure impairs nutrient diffusion and reduces the metabolic activity of intervertebral disc cells, potentially leading to cell death. In this study, the VG exhibited lower maximum stress in the L1–L2 disc during flexion, extension, and lateral bending but higher stress during rotation. This may be attributable to the mechanical interaction between the posterior spinal elements, particularly the facet joints. During rotation, the facet joints bear the majority of the torsional load and restrict motion. When bone cement is distributed symmetrically in a vertical configuration, it most effectively restores the stiffness and load-bearing capacity of the anterior and middle columns. Consequently, a greater portion of the rotational torque is transferred to and absorbed by the posterior elements, including the facet joints. In contrast, an inclined cement distribution may partially disrupt uniform load transfer in the anterior column, thereby altering torque transmission across the motion segment. This redistribution may reduce the load borne by the facet joints and subsequently decrease the shear stress transmitted to the posterior annulus fibrosus during rotation.In the L2–L3 disc, the VG exhibited lower maximum stress than both the FG and IG, suggesting that vertical cement distribution can alleviate intervertebral disc stress. Nevertheless, it is advisable to limit rotational activities during postoperative rehabilitation to mitigate the risk of accelerated disc degeneration.

This study has several limitations. First, in assessing vertebral stability after cement augmentation, the material properties of cortical bone, cancellous bone, and soft tissues were simplified as isotropic linear elastic materials. Although this assumption may influence absolute stress and displacement values, it has been widely adopted and validated in comparative biomechanical analyses of spinal implants. For example, Zhou et al. (1) applied a similar linear elasticity model to evaluate cement distribution patterns and successfully identified biomechanical distinctions between surgical strategies. Second, the trabecular bone exhibits heterogeneous density and irregular fracture propagation, resulting in variable cement morphologies in clinical practice. To standardize model construction and ensure reproducibility, this study adopted a simplified cylindrical representation of cement, a method commonly used in finite element modeling. Dai et al. (26) reported that variations in cement morphology may slightly affect localized stress fields but do not alter the overall comparative conclusions regarding different filling techniques. This finding is consistent with Rohlmann et al. (41), who demonstrated that cement volume is the dominant factor affecting maximum vertebral stress, whereas cement morphology exerts a relatively minor influenceFirst, in assessing vertebral stability after cement augmentation, the material properties of cortical bone, cancellous bone, and soft tissues were simplified as isotropic linear elastic materials. Although this assumption may influence absolute stress and displacement values, it has been widely adopted and validated in comparative biomechanical analyses of spinal implants. For example, Zhou et al. (1) applied a similar linear elasticity model to evaluate cement distribution patterns and successfully identified biomechanical distinctions between surgical strategies. Second, the trabecular bone exhibits heterogeneous density and irregular fracture propagation, resulting in variable cement morphologies in clinical practice. To standardize model construction and ensure reproducibility, this study adopted a simplified cylindrical representation of cement, a method commonly used in finite element modeling. Dai et al. (26) reported that variations in cement morphology may slightly affect localized stress fields but do not alter the overall comparative conclusions regarding different filling techniques. This finding is consistent with Rohlmann et al. (41), who demonstrated that cement volume is the dominant factor affecting maximum vertebral stress, whereas cement morphology exerts a relatively minor influence. Finally, finite element analysis inherently simplifies physiological conditions, as vertebrae are subjected to complex, multidirectional forces during daily activities. Therefore, while the model offers valuable biomechanical insights, it cannot fully replicate the multifactorial loading environment experienced *in vivo*, which may limit the precision of clinical extrapolation.

## Conclusion

Using finite element analysis, this study compared the biomechanical behavior of vertical (VG) and inclined (IG) bone cement distribution patterns. The results demonstrated that the VG configuration provides superior stabilization of fractured vertebrae and achieves a more balanced distribution of stress across the vertebrae and intervertebral discs. These findings suggest that vertical cement distribution offers a biomechanically advantageous strategy for vertebral augmentation and may serve as a valuable reference for optimizing surgical techniques in clinical practice.

## Data Availability

The original contributions presented in the study are included in the article/Supplementary Material, further inquiries can be directed to the corresponding author.
